# Interplay between *P. gingivalis, F. nucleatum* and *A. actinomycetemcomitans* in murine alveolar bone loss, arthritis onset and progression

**DOI:** 10.1038/s41598-018-33129-z

**Published:** 2018-10-11

**Authors:** Meinolf Ebbers, Paul M. Lübcke, Johann Volzke, Katja Kriebel, Cathleen Hieke, Robby Engelmann, Hermann Lang, Bernd Kreikemeyer, Brigitte Müller-Hilke

**Affiliations:** 1 Institute for Immunology, University Medical Center Rostock, Rostock, Germany; 2 Department of Operative Dentistry and Periodontology, University Medical Center Rostock, Rostock, Germany; 3 Institute of Medical Microbiology, Virology and Hygiene, University Medical Center Rostock, Rostock, Germany; 4 Present Address: Microbiology, Institute for Biosciences, University of Rostock, Rostock, Germany

## Abstract

Increasing evidence supports the association of periodontitis with rheumatoid arthritis. Even though a prominent role has been postulated for *Porphyromonas gingivalis*, many bacterial species contribute to the pathogenesis of periodontal disease. We therefore investigated the impact of *Porphyromonas gingivalis* as well as other major pathobionts on the development of both, periodontitis and arthritis in the mouse. Pathobionts used - either alone or in combination - were *Porphyromonas gingivalis*, *Fusobacterium nucleatum* and *Aggregatibacter actinomycetemcomintans*. Periodontitis was induced via oral gavage in SKG, DBA/1 and F1 (DBA/1 × B10.Q) mice and collagen-induced arthritis was provoked via immunization and boost with bovine collagen type II. Alveolar bone loss was quantified via micro computed tomography, arthritis was evaluated macroscopically and histologically and serum antibodies were assessed. Among the strains tested, only F1 mice were susceptible to *P. gingivalis* induced periodontitis and showed significant alveolar bone loss. Bone loss was paralleled by antibody titers against *P. gingivalis*. Of note, mice inoculated with the mix of all three pathobionts showed less alveolar bone loss than mice inoculated with *P. gingivalis* alone. However, oral inoculation with either *F. nucleatum* or *A. actinomycetemcomintans* alone accelerated subsequent arthritis onset and progression. This is the first report of a triple oral inoculation of pathobionts combined with collagen-induced arthritis in the mouse. In this interplay and this particular genetic setting, *F. nucleatum* and *A. actinomycetemcomitans* exerted a protective impact on *P. gingivalis* induced alveolar bone loss. By themselves they did not induce periodontitis yet accelerated arthritis onset and progression.

## Introduction

Rheumatoid arthritis (RA) is a chronic autoimmune disease that primarily affects the joints but can turn into a systemic disorder involving the heart, the eyes, the gastrointestinal tract, the lungs, the kidneys and the nervous system^1,2^. Untreated, RA will lead to irreversible damage and functional loss of the joints and to comorbidity^3^. This disease is multifactorial with genetic as well as environmental factors triggering its pathogenesis^4^. Although intensively studied, the exact mechanisms of disease development in RA are still unknown^5^. As there is no available cure, an early diagnosis and onset of treatment are mandatory in order to avert increasing pain for the patients and preserve function and quality of life^6^.

Some risk factors for RA have been identified, among them periodontitis (PD)^7^. PD results from dysbiosis of the oral microbiota with plaque accumulating at the gingival margin, development of gingivitis, the failure of the immune system to eradicate the microbial challenge and subsequent progression to chronic PD including the loss of alveolar bone^8,9^. However, just like RA, PD has a complex pathology and multifactorial etiology^10^. Various oral pathobionts have been found associated with destructive PD, among them members of the early colonizing green complexes, members of the bridging orange complex and members of the late colonizing red complex^11^. While the observation of arthritis being associated with gum disease has already been described in the 19^th^ century, the exact nature of this relationship remains enigmatic^12^.

Recent explanations for the association of RA with PD include specific immune modulations and altered gut microbioms due to oral dysbiosis^13^. However, research into this association gained further momentum by two observations, the first one being that antibodies against citrullinated peptide antigens (ACPA) are highly specific for RA and precede the onset of the disease by several years^14^ and second, that *P. gingivalis* is so far the only oral pathobiont that was proven to be capable of citrullination^15^. Since then, major research effort was placed on *P. gingivalis* and the peptidylarginine deiminases (PAD) catalyzing citrullination^1,7^. However, as the oral cavity is home to several hundred different bacterial species, it is difficult to attribute specific functions to single pathobionts in the human setting^16^. We therefore turned to a mouse model and by inoculating mice with three different oral pathobionts created an experimental setting that approximates periodontal dysbiosis in patients. The oral pathobionts we chose were *P. gingivalis, F. nucleatum* and *A. actinomycetemcomitans*^10,17,18^. *P. gingivalis* is considered the major etiologic agent contributing to chronic periodontitis and is reliably used in animal models for periodontitis^19^. While *F. nucleatum* and *A. actinomycetemcomitans* have been associated with aggressive periodontitis, there is as of yet no data confirming a relevant role for *F. nucleatum* in the etiology of joint diseases. As a member of the bridging complex in between early and late oral colonizers, it may though indirectly be involved in the pathogenesis of RA^20,21^. Moreover, *F. nucleatum* is associated with cardiovascular disease which in turn is a comorbidity of RA^22^. Interestingly, *A. actinomycetemcomitans* was found to induce hypercitrullination in human host neutrophils and thus also qualifies as a cause of autoantigen formation in RA^23,24^. The objective of this study was to examine the impact of three different oral pathobionts – either alone or in combination – on the development and progression of PD as well as arthritis. To that extent, we chose mouse strains that either spontaneously develop arthritis (SKG)^25^ or were susceptible (DBA/1 × B10.Q F1) and resistant (DBA/1) to collagen-induced arthritis (CIA), respectively. We postulated that (i) PD as a consequence of oral inoculation with a certain pathobiont is dependent on the genetic background, (ii) the combination of three different and aggressive pathobionts will aggravate PD and (iii) the more severe the preexisting PD, the more exacerbated the subsequent arthritis.

## Results

### DBA/1J × B10.Q F1 mice were most susceptible to *P. gingivalis* induced periodontitis and anti-*Pg* antibody response

**Figure 1:**
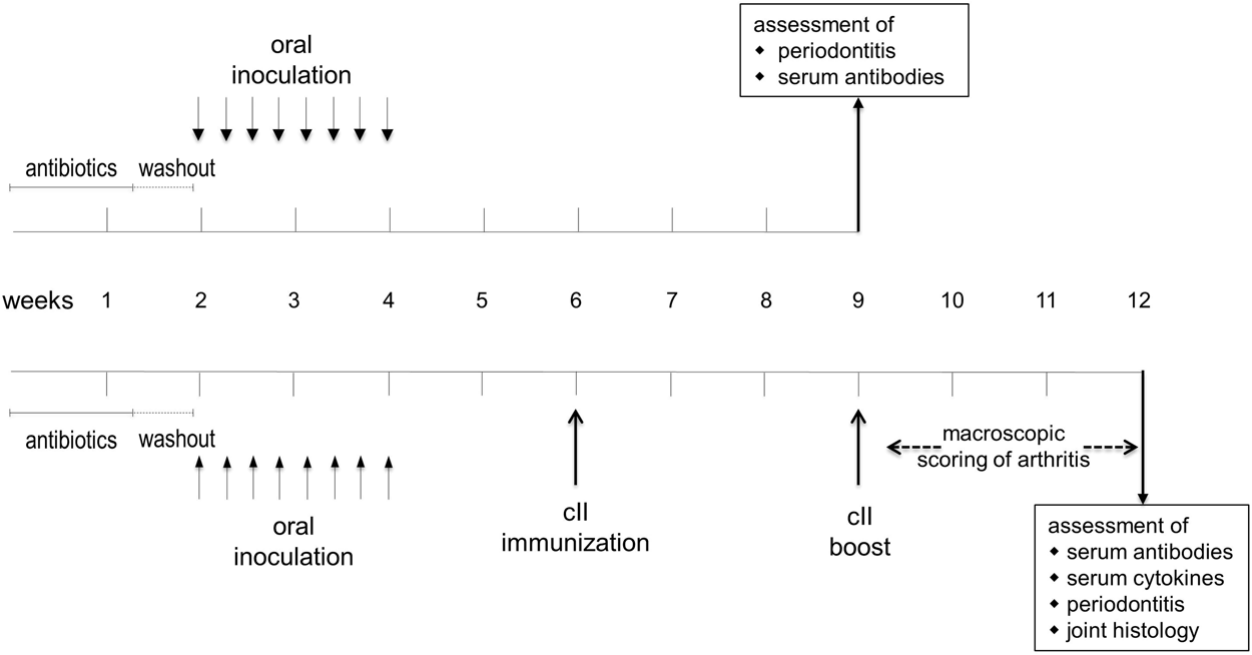
The experimental setup. For the induction of PD an initial antibiotic treatment followed by a washout period and eight oral inoculations with pathobionts was performed. In the absence of subsequent arthritis induction (upper diagram), mice were monitored for five weeks before they were sacrificed, serum assessed for antibodies and mandibles for alveolar bone loss. Arthritis induction (lower diagram) included a primary immunization with bovine collagen type II and a boost three weeks thereafter. Macroscopic signs of arthritis were monitored until the mice were sacrificed. Mandibles were assessed for alveolar bone loss, joints scored histologically and serum tested for inflammatory cytokines and antibodies.

**Figure 2:**
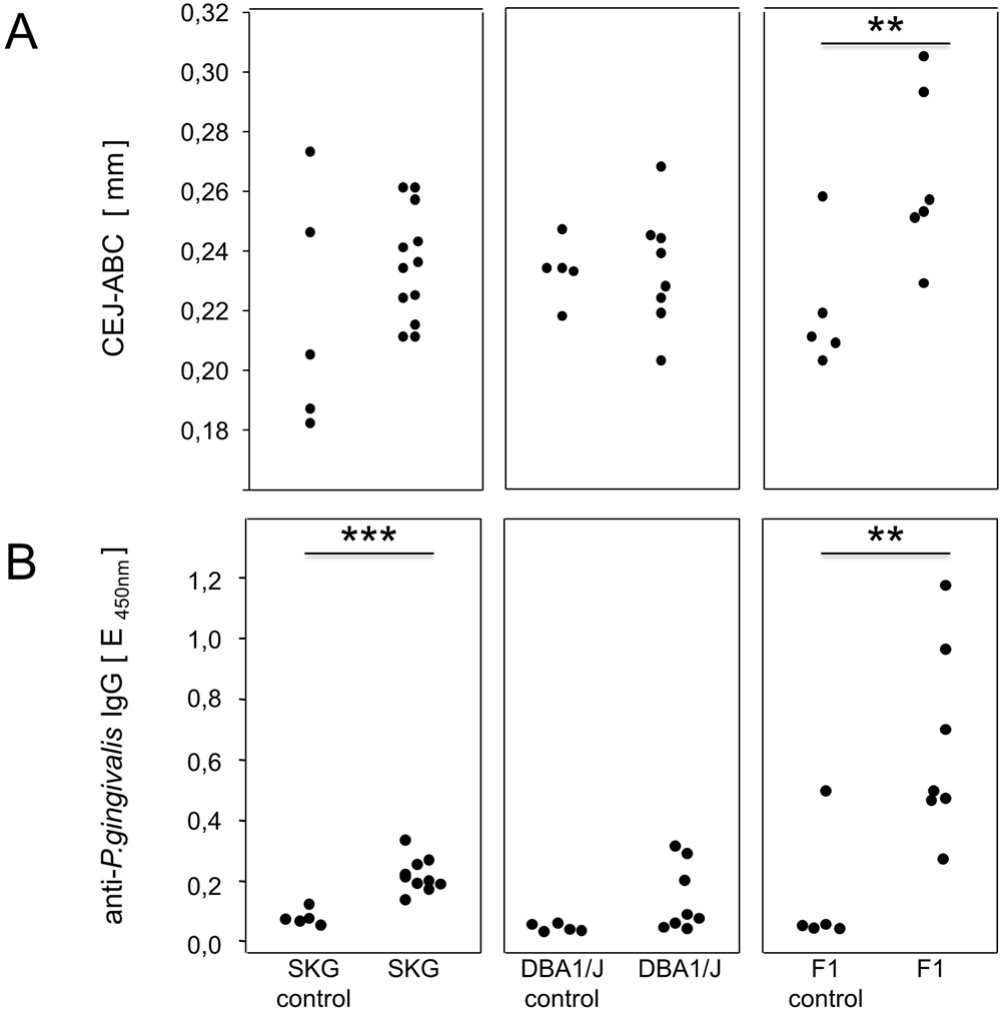
DBA/1J × B10.Q F1 mice were most susceptible to *P. gingivalis* (*Pg*) induced PD and anti-*Pg* antibody response. (**A**) Depicts the alveolar bone loss after PD induction by presenting the distance from the cementoenemal junction to the alveolar bone crest (CEJ-ABC) in millimeters of each individual mouse that was orally inoculated and their control mates. Assessment was performed by µCT. (**B**) Shows the anti-*P. gingivalis* antibody titers comparing again individual mice that were orally inoculated to their untreated controls. Numbers of mice per group were SKG N = 10, DBA/1J N = 8, F1 (DBA/1J × B10.Q) N = 7, controls were N = 5 for each strain.

The objective of the first experiment was to determine the most susceptible mouse strain for the induction of periodontitis (PD) via oral inoculation with *P.gingivalis* (*Pg*). To that extent, ten SKG, eight DBA/1 and seven DBA/1 × B10.Q F1 mice were orally inoculated with *P. gingivalis*. Five weeks after the final oral inoculation, mice were sacrificed and the mandible specimens were collected and imaged via micro computed tomography (µCT) (Fig. 1 and Supplementary Appendix). PD was assessed by measuring the distance from the cemento-enamel junction (CEJ) to the alveolar bone crest (ABC) and calculating the bone loss by comparing the *P. gingivalis*-inoculated mice to their untreated controls (n = 5 per strain). The results in Fig. 2A are thus displayed in millimeters following *P. gingivalis* inoculation and show that the most severe bone loss occurred in F1 (DBA/1J × B10.Q) mice with a mean 1.2-fold increase in the CEJ-ABC distance compared to their untreated controls. In contrast, there was significantly less bone loss in SKG (1.1-fold) and none in DBA/1 (1.0-fold) mice. The differences between the treated F1 mice compared to their untreated controls were statistically significant on the one hand. On the other, no differences were observed between treated SKG or DBA/1 mice and their untreated controls (Fig. 2A). In summary, F1 mice were most susceptible to the induction of PD and were therefore chosen for all follow-up experiments.

To rule out that the absence of bone loss in SKG and DBA/1J mice resulted from a lack of exposure to oral pathobionts, serum antibodies to *P. gingivalis* were quantified as proxy. As shown in Fig. 2B, SKG and DBA/1 mice displayed 2.7- and 3.0-fold increase in anti-*P. gingivalis* titers confirming bacterial contact. Of note, F1 (DBA/1J × B10.Q) mice showed a 13-fold increase in anti-*P. gingivalis* antibody titers and therefore were not only most susceptible to the induction of PD but also able to mount the most efficient anti-*P. gingivalis* response.

### *P. gingivalis* induced the most severe alveolar bone loss

**Figure 3:**
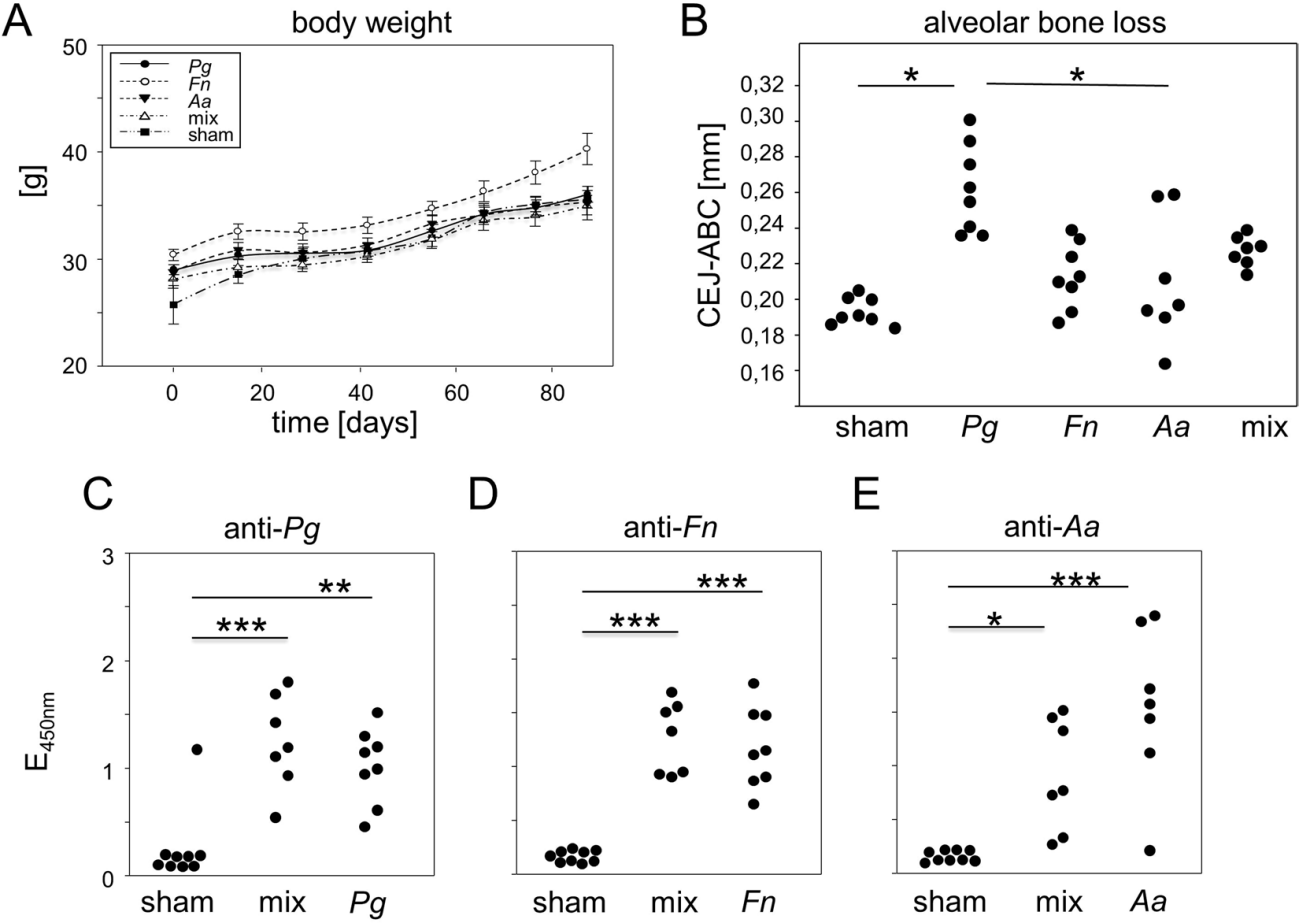
*P. gingivalis* induced the most severe alveolar bone loss. (**A**) During the experimental period, all mice developed physiologically and gained weight in a comparable fashion. (**B**) Dot plots present the distance from the CEJ-ABC of orally inoculated or sham treated F1 (DBA/1J × B10.Q) mice. (**C**–**E**) Plots represent antibody titers against oral pathobionts. *Pg*: *P. gingivalis; Fn: F. nucleatum; Aa*: *A. actinomycetemcomitans*; mix: mice were orally inoculated with a mix of all three pathobionts or just sham treated. Numbers of mice used per group were *Pg* N = 8, *Fn* N = 8, *Aa* N = 7, mix N = 7, sham N = 9.

The next set of experiments served to compare the PD inducing potentials of *P. gingivalis*, *F. nucleatum and A. actinomycetemcomitans*, either alone or in combination. To that extent, F1 (DBA/1J × B10.Q) mice were pretreated with antibiotics and were then inoculated with 2 × 10^9^ cfu of either of the bacterial species or a mix containing 2 × 10^9^ cfu of each sticking to the previously mentioned application protocol (Fig. 1). In order to rule out that PD impacted on the physiological development of mice, animals were weighed regularly. The weight curves are displayed in Fig. 3A and confirm that all mice inoculated with oral pathobionts developed comparably to sham treated mice. At the end of the observation period, mice were sacrificed, the mandibles were collected and imaged via µCT as decribed above. Quantification of the distance between CEJ and ABC showed that both, *F. nucleatum and A. actinomycetemcomitans* were significantly less potent in inducing bone loss than *P. gingivalis* and yielded hardly any differences from the sham treated controls. Importantly, mixing *P. gingivalis* with *F. nucleatum* and *A. actinomycetemcomitans* resulted in less bone loss than inoculating with *P. gingivalis* alone, even though the comparison to sham treated mice was still significant. Figure 3B presents the increase in bone loss in orally inoculated mice over their sham treated controls and compares single vs. triple inoculations. Note that longer observation periods led to more bone loss (Compare Figs 2A and 3B). Our results thus show that inoculating mice with *P. gingivalis* alone led to the most severe alveolar bone loss.

In order to assess whether individual bacterial species in a mix would impact on the immune system to a comparable extent as the same species infected alone, we quantified the serum antibodies against these pathogens as proxy. Figure 3C–E show that indeed, all mice inoculated showed elevated anti-bacterial antibody titers compared to their sham treated controls yet the heights of the titers were independent of single or mixed inoculations.

### Oral inoculations with *F. nucleatum* or *A. actinomycetemcomitans* accelerated the onset and progression of subsequent collagen induced arthritis

We next assessed whether PD or the exposure to oral pathobionts would impact on the course of subsequent arthritis. We therefore provoked collagen-induced arthritis in the F1 (DBA/1J × B10.Q) mice that had previously been inoculated with *P. gingivalis*, *F. nucleatum* and *A. actinomycetemcomitans* either alone or in the mix or had been sham treated. In detail, mice were immunized with bovine collagen type 2 in complete Freund’s adjuvant two weeks after the final oral inoculation and were boosted three weeks thereafter (Fig. 1). Macroscopic scoring of the hind and fore limbs was carried out every third day beginning three days after the boost. At the end of the observation period, arthritis incidence and scores were compared as were serum cytokines and antibodies against *P. gingivalis*, *F. nucleatum* and *A. actinomycetemcomitans* as well as ACPA.

**Figure 4:**
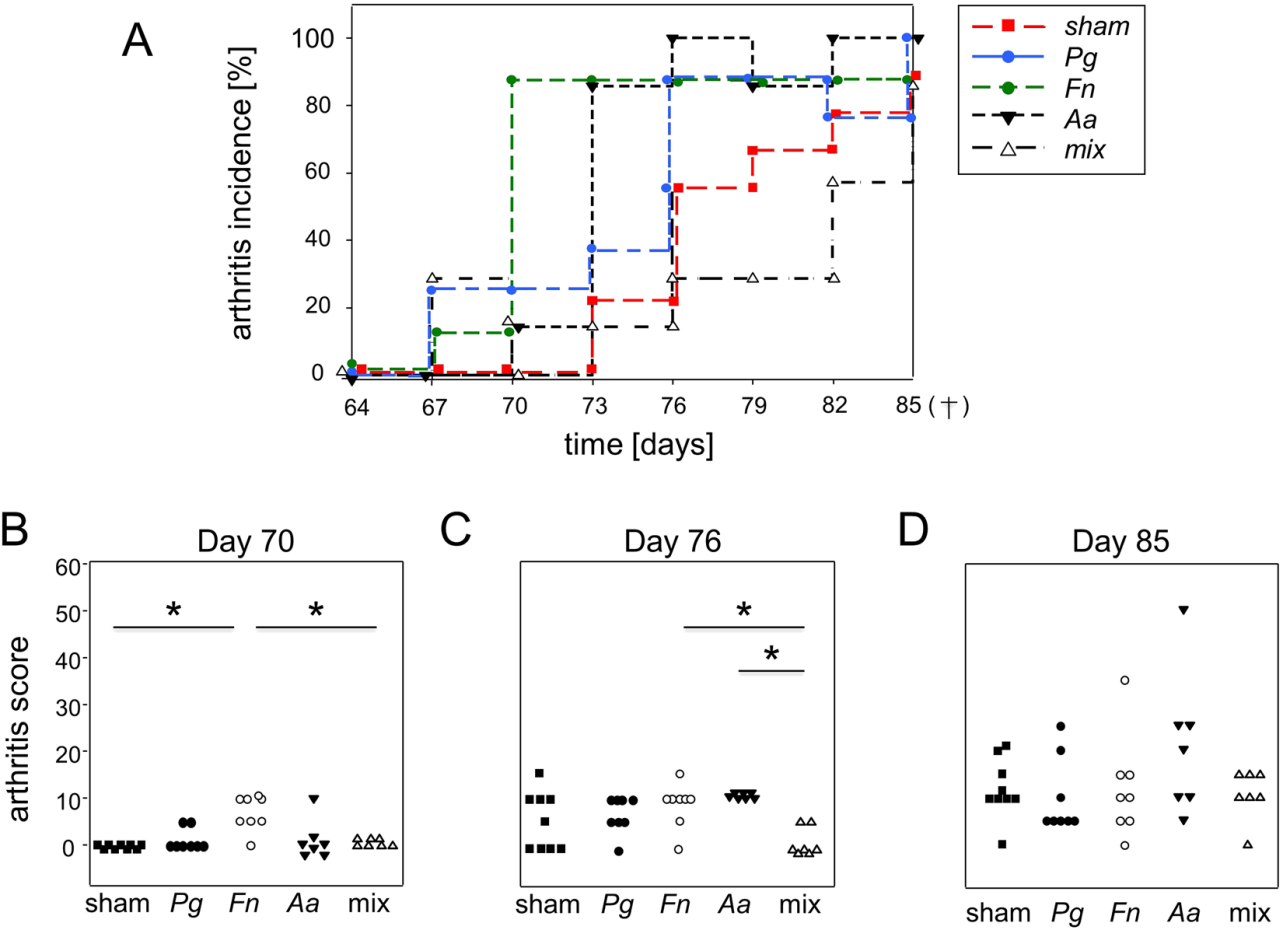
Oral inoculations with *F. nucleatum* or *A. actinomycetemcomitans* accelerated the onset and progression of subsequent CIA. (**A**) The plot shows the incidence of arthritis over the experimental period and compares mice orally inoculated with *P. gingivalis* (Pg), *F. nucleatum* (Fn), *A. actinomycetemcomitans* (Aa), the mix of all three species (mix) or sham treated controls (sham). (**B**–**D**) Display representative individual macroscopic arthritis scores at experimental day 70, 76 and at the end of the experimental period before sacrifice.

Cumulative incidences of arthritis at the end of the observation period were 85.7% for the mix treated group, 87,5% for the group treated with *F. nucleatum*, 88,9% for sham treated animals and 100% for the *A. actinomycetemcomitans* and *P. gingivalis* treated groups (Fig. 4A). However, the groups differed significantly with respect to the onset and progression of the disease. While mice pre-inoculated with the mix of *P. gingivalis*, *F. nucleatum* and *A. actinomycetemcomitans* showed the slowest rise in incidence, mice pre-inoculated with either *F. nucleatum* or *A. actinomycetemcomitans* fell ill with arthritis the fastest. A Log Rank (Mantel-Cox) test was performed in order to compare the differences in incidence at the late experimental period. As expected, the P value (0.061) indicated some between-groups-difference yet did not reach statistical significance. In contrast, performing a Breslow (generalized Wilcoxon) or Tarone-Ware test in order to assess the differences during the early and middle experimental period respectively yielded statistically significant differences with P values of 0.034 and 0.039.

**Figure 5:**
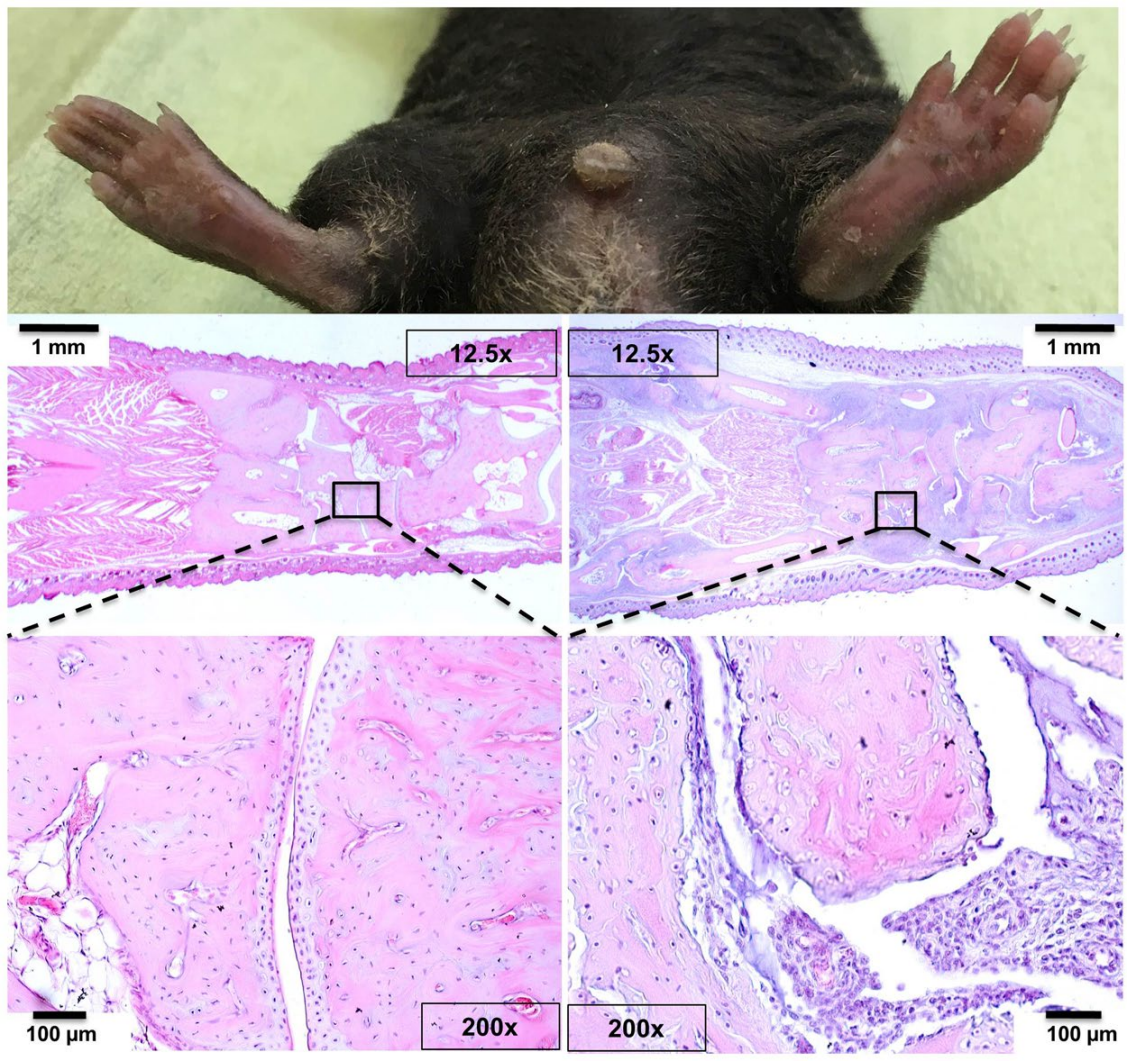
Macroscopic picture and HE-stained thin sections of non-inflamed (left panels) and inflamed (right panels) hind paws. The lower insert shows massive leukocyte infiltrations into the metatarsal joint spaces and illustrates the onset of articular cartilage destruction. 12,5x (middle) and 200x (lower) magnifications.

Macroscopic arthritis scores were assessed every third day beginning three days after the boost. Statistically significant differences occurred temporarily at experimental days 70 and 76 (Fig. 4B,C). At these time points, mice pre-inoculated with *Fn* and *Fn* or *Aa* respectively, showed the highest scores. At the end of the observation period, mice pre-inoculated with *Aa* still showed the highest arthritis scores with a mean of 20.7 while the *Pg* treated group was least effected. However, there were no more statistically significant differences between groups (Fig. 4D). Figure 5 presents an example of an inflamed hind limb and its massive leukocyte infiltrations. Titers of potentially pathogenic antibodies against bovine and murine collagen type II turned out comparably between groups (Supplementary Appendix Fig. 2B,C). Moreover, there was no ACPA production above background, confirming results from Stoop *et al*. showing that mice do not produce ACPA (Supplementary Appendix Fig. 2A)^26^. Likewise, the serum cytokines IL-1α, IL-1β, IL-6, IL-10, IL-12p70, IL-17A, IL-23, IL-27, GM-CSF, IFNβ, IFNγ, MCP-1 and TNFα did not show any between group differences at this late time point (Supplementary Appendix Fig. 3).

## Discussion

The first set of experiments served to identify a susceptible mouse strain for the induction of periodontitis and bone loss. We had postulated upfront that periodontitis (PD) following oral inoculation with *P. gingivalis* is dependent on a susceptible genetic background. This hypothesis turned out to be true as only F1 (DBA/1 × B10.Q) mice showed a significant alveolar bone loss compared to their untreated controls. Interestingly, the parental DBA/1 mouse did not show any signs of PD. This finding is in line with a previous publication by Maresz and coworkers who showed that DBA/1 mice are resistant to oral colonization with *P. gingivalis*^27^. In contrast, Marchesan and colleagues showed a significant alveolar bone loss following oral *P. gingivalis* inoculation^28^. These conflicting data may be explained by minor genetic drifts in the various labs or alternatively by different microbioms impacting on the immune responses. The third mouse strain, SKG, is prone to spontaneous arthritis due to an altered T cell receptor signaling and was included to show whether periodontitis would impact on spontaneous autoimmunity^25^. However, SKG mice in our experiments did neither exert alveolar bone loss nor did they develop arthritis. This was surprising as *P. gingivalis* was reported to trigger the TLR2 pathway which in turn was described to promote spontaneous arthritis in SKG mice^29^. Likewise, i.p. injection of *P. gingivalis* into SKG mice was reported to exacerbate arthritis. In remains possible though that different routes of inoculation lead to conflicting results^30^. In summary, the comparison of three different mouse strains confirmed the notion that PD is a multifactorial disease requiring not only oral pathobionts and appropriate environmental conditions, but also a suitable genetic predisposition. Of note, we have repeatedly observed that F1 (DBA/1 × B10.Q) mice are more susceptible to collagen-induced arthritis than the parental DBA/1 mice and were therefore intrigued to find the same is true for PD. Most importantly, the antibody response to *P. gingivalis* was also most pronounced in the F1 mice. Even though we cannot rule out that the various antibody responses simply reflect different numbers of pathobionts colonizing the oral cavity, we favor the notion that these antibodies are related to the pathology of PD – either directly by routing complement activity to the periodont or indirectly by reflecting a systemic T helper cell response.

The second hypothesis triggering our experiments postulated that oral inoculation with a mix of three different pathobionts – one from the green, one from the orange and one from the red complex - would exacerbate PD induced by a single pathobiont alone^11,17,31^. Of note, when comparing single inoculations with *P. gingivalis* (member of the red complex) to *F. nucleatum* (member of the bridging orange complex) to *A. actinomycetemcomitans* (member of the early colonizers or green complex), the most severe bone loss was observed following inoculation with *P. gingivalis*. These data are unexpected and contradict previous publications showing that in terms of alveolar bone loss induction, *F. nucleatum* and *P. gingivalis* synergize and that *F. nucleatum* is superior to *P. gingivalis*^32–34^. We can only speculate that again genetic backgrounds play a role here. As we only tested *P. gingivalis* for its potential to induce periodontitis in various mouse strains, we cannot exclude the possibility that F1 mice are particularly resistant or that DBA/1 or SKG mice are particularly susceptible to *F. nucleatum* and *A. actinomycetemcomitans* induced periodontitis. Alternatively, the three days of experimental wash-out following the initial antibiotics therapy may have sufficed to reestablish a biofilm of naturally occurring early and bridging complexes, preventing *F. nucleatum* and *A. actinomycetemcomitans* from efficiently colonizing the oral cavities. Being a late colonizer may simply have been advantageous for *P. gingivalis*. In fact, *in vitro* experiments showed that the attachment and invasion of *P. gingivalis* was reduced compared to *F. nucleatum* and *A. actinomycetemcomitans*^35^. Furthermore, a coinfection of these species resulted in an enhanced attachment and invasion to human gingival epithelial cells if the bacteria were administered separately^36^. Analyzing the oral microbiome immediately after cessation of the antibiotics therapy will in the future help to elucidate these early steps of recolonization.

Our most important observation was that a mixed inoculation resulted in less bone loss compared to an inoculation with *P. gingivalis* alone. We cannot formally rule out displacement of one pathobiont by the others however, our antibody titres confirm that independent of a mixed or single inoculation, all bacteria survived long enough to trigger comparable antibody responses. Future experiments will therefore need to show if *F. nucleatum* and *A. actinomycetemcomitans* indeed mediate protective effects. Of note, co-inoculation of *P. gingivalis* with *F. nucleatum* was previously shown to have a different impact on the immune response than inoculation with *P. gingivalis* alone and led to an isotype shift in the pathobiont-specific antibodies^37^. Likewise, various *P. gingivalis* strains were shown to elicit different cytokine responses in C56BL/6 mice^38^. As of yet, we have not observed different titers of pathobiont-specific antibodies following triple or single inoculations however, a detailed analysis of the various isotypes is still lacking. Importantly, our experimental set-up paves the way for future studies exploring the *in vivo* interplay of multiple bacteria species.

Our third hypothesis postulated that a preexisting PD would exacerbate subsequent CIA. Several studies in the past investigated the effect of preexisting periodontitis on collagen-induced arthritis in mice^27,39,40^. We here evaluated arthritis twofold, via the incidence of arthritis over the full observation period and via the individual macroscopic scores at endpoint. While we did not observe any differences in the end point macroscopic scores comparing sham treated mice to mice inoculated with pathobionts, there were significant differences evaluating the incidence. Astoundingly, even though there were hardly any signs of PD, the fastest onset of arthritis was observed for mice inoculated with *F. nucleatum* and *A. actinomycetemcomitans*. In contrast, mice that had most severe PD following inoculation with *P. gingivalis* alone showed an arthritis onset that was comparable to sham treated mice. Interestingly, mice inoculated with the mix of *P. gingivalis, F. nucleatum* and *A. actinomycetemcomitans* that developed only mild periodontitis, showed the slowest onset of arthritis and therefore again suggested an ameliorating effect of the mix.

While we cannot explain our observations on a molecular level yet, we can only speculate that it is neither PD nor alveolar bone loss per se that aggravate subsequent arthritis. We rather assume that oral pathobionts modulate the systemic immune response and - depending on whether they act alone or in concert - lead to a cytokine milieu that may either be protective or supportive for subsequent disease induction. Along these lines, oral pathobionts were previously shown to modulate the gut microbiome as well as systemic cytokines^41,42^. The immunological fine-tuning provoked by the various oral pathobionts remains enigmatic though as both, PD as well as CIA depend on dominating Th17 responses^43^. Unfortunately, our analysis of inflammatory cytokines at endpoint did not allow for a detailed differentiation of pathobiont induced immune responses. Likewise, at endpoint we did not observe any differences in the titers of pathogenic antibodies recognizing collagen type 2 or citrullinated peptide antigens^44^.

There are limitations to our study that need to be pointed out: The most severe limitation is the restricted observation period. Earlier time points and cytokine measurements throughout the observation period are required to evaluate the various immune responses elicited by the pathobionts. And as the symptomatic onset of arthritis has been described to vary widely, later time points are required in order to not miss pathobiont specific late effects. We also did not formally attribute alveolar bone loss to bacterial infection as we did not re-isolate live bacteria from periodontal lesions. And even though we did prove contact with pathobionts via specific serum antibodies, contact may have occurred anywhere along the oral and gastrointestinal mucosa. We therefore cannot rule out that oral pathobionts induced intestinal inflammation and citrullination which may have systemically triggered alveolar bone loss. However, we did not observe any correlation between ACPA titres and alveolar bone loss (data not shown).

We here combined for the first time three major oral pathobionts in an *in vivo* study with CIA and showed that even though the development of PD and arthritis were not necessarily paralleled, the pathobionts exerted specific impacts on both, alveolar bone loss and arthritis onset and progression.

## Materials and Methods

### Mice

DBA/1 and B10.Q mice were originally purchased from Harlan Winkelmann (Borchen, Germany) and SKG mice were originally obtained from Shimon Sakaguchi^45^. Mice were subsequently bred in our animal care facility under specific germfree conditions, housed in cages with a 12 hours light/dark cycle and given water and food ad libitum. All mice entering the experiments were between 8 and 10 weeks of age. The local state’s animal care committee (Landesamt für Landwirtschaft, Lebensmittelsicherheit und Fischerei M-V; www.lallf.de) approved all experiments (7221.3-1.1-052/14) and all experiments were carried out in accordance with the relevant guidelines and regulations.

### Bacterial strains

Bacterial strains - *Porphyromonas gingivalis* W83 (Institute of Medical Microbiology, Virology and Hygiene, University Medical Center Rostock, Rostock, Germany) and *Fusobacterium nucleatum* (American Type Culture Collection, Manassas, USA, ATCC 25586) were purchased from commercial providers. Both species were grown in an anaerobic atmosphere (10% CO_2_, 10% H_2_, 80% N_2_) using Pepton-Yeast-Glucose (PYG) medium, which was supplemented with 5 ug/ml hemin and 1% vitamin K solution. The species *Aggregatibacter actinomycetemcomitans* (DSMZ, Braunschweig, Germany, DSMZ 11123) was cultured in brain heart infusion medium (BHI, Invitrogen, Carlsbad, CA, USA) at 37 °C under a 5% CO_2_-ambient atmosphere. All bacterial species were grown until they reached mid-logarithmic phase. The time point for cell harvest was variable according to medium- and growth condition- specific growth rates µ [h^−1^] and doubling times t_d_ [min], which were determined in preliminary experiments for all species. Bacterial cultures were centrifuged, washed with PBS and resuspended in DMEM medium to a cell number of 2 × 10^9^ cells per 50 µl. Aliquots of 2 × 10^9^ cfu were kept frozen at −80 °C until immediately before the respective experiments. For inoculation, aliquots were thawed and the medium removed. 2 × 10^9^ cfu were then suspended in 50 µl PBS containing 2% sodium carboxymethylcellulose (Sigma-Aldrich, St. Louis, MO, USA). To confirm viability and to perform dose adjustment if necessary, aliquots were checked weekly by plating the thawed bacteria and counting the CFU.

### Induction of periodontitis

The experimental setup is displayed in Fig. 1. In preparation for the induction of periodontitis (PD), mice initially received a 10 days course of antibiotics administered via the drinking water containing 2% of antibiotics (Cotrim K - ratiopharm 240 mg/5 ml, Ratiopharm, Ulm, Germany) followed by a 3 days washout period. For induction of PD, mice were anesthetized with intraperitoneal injections of 0.75 mg Esketamin (100 mg/ml, bela-pharm, Vechta, Germany)/0.05 mg Xylazin (20 mg/ml, Bayer AG, Leverkusen, Germany) per 10 g of body weight. Inoculation of bacteria was then performed via oral gavage with the prepared aliquots immediately within one hour after thawing process. After gavage, mice were left under visual monitoring underneath a heat lamp for one hour without access to food or water. Oral inoculation was repeated seven times every other day following the first gavage. Sham treated mice received oral gavage with 50 µl PBS containing 2% sodium carboxymethylcellulose but no bacteria. Throughout the first four weeks of the experiment, mice were weighed every other day and every third day thereafter.

### Induction of CIA

The induction of collagen-induced arthritis (CIA) was performed as described previously^46^. Briefly, on experimental day 42, mice were immunized by injecting 140 µg bovine type II collagen (mdbioscience, Egg b. Zürich, Switzerland) in 0.1 M acetic acid emulsified in an equal amount of complete Freund’s adjuvant (CFA, Becton, Dickinson and Company, Franklin Lakes, NJ, USA) subcutaneously at both sides of the base of the tail. Three weeks after the primary immunization, mice were boosted by injecting 140 µg bovine type II collagen in 0.1 M acetic acid emulsified in an equal amount of incomplete Freund’s adjuvant (IFA). Control mice were left untreated.

### Assessment of alveolar bone loss

At the end of the experiments, mice were sacrificed under deep anesthesia via cervical dislocation. Mandibles were carefully dissected and fixed in 4% paraformaldehyde (PFA) for 7 days, washed with tap water for 0.5 h, stored in NaCl 0.9% and then used for imaging. Three-dimensional microcomputed tomography images were obtained using a SkyScan 1076 micro-CT scanner (Bruker, Billerica, MA, USA). The following parameters for scanning were used: resolution of 9 µm, Al 0.5 mm filter, rotation step 0.5 °, averaging frames 3, source voltage 48 kV, source current 200 µA. In accordance with the application of bacteria, only the right hemimandibles were examined. Analyses were done in millimeters, assessing the distance from the cementoenemel junction (CEJ) to the alveolar bone crest (ABC) at three sites each at the lingual and buccal sides of each molar, totaling 18 sites (Supplementary Appendix Fig. 1). For a given animal, all values were collected and means calculated.

### Macroscopic scoring of arthritis and histological examination

Macroscopic scoring of the hind and fore limbs was carried out every third day beginning three days after the boost. Scoring was performed according to the following scheme: 0 = no signs of arthritis, 1 = redness and/or swelling for each affected digit, 5 = redness and/or swelling for each affected paw area, 5 = redness and/or swelling for each wrist joint. A maximum score of 60 per mouse and scoring day can thus be reached. All animals showing macroscopic signs of joint inflammation (i.e. redness and/or swelling) were considered diseased and were evaluated for disease incidence.

After the mice were sacrificed at the end of the experiments, paws were removed and fixed in formalin 4% for two weeks, washed with tap water for 30 minutes and then decalcified using Usedecalc (Medite, Burgdorf, Germany). After one week, samples were washed again with tap water and then embedded into paraffin. Serial sections of 5 µm thickness were obtained in longitudinal orientation, mounted on slides and stained with hematoxylin and eosin (HE). Histological sections were imaged using a 12.5x and 200x magnification, respectively (Axioplan 2, Zeiss, Oberkochen, Germany).

### Serum analysis

At the end of the experiments, roughly 1,5 ml of blood per mouse was obtained from the orbital vein plexus under deep anesthesia. The serum was collected and stored at −20 °C for further processing. ACPA IgG levels were measured by combining CCP2- (Euroimmun, Lübeck, Germany) and MCV- (Orgentec, Mainz, Germany) coated ELISA plates with an anti-mouse IgG antibody coupled to horse radish peroxidase (STAR13B; Bio-Rad Laboratories, Hercules, CA, USA) as described before^47^. In short, the sera were applied at a dilution of 1:50 and 1:200 for 1.5 h at RT for the CCP- and MCV-coated ELISA plates, respectively. Thereafter, the plates were incubated with the detection antibody at a dilution of 1:1000 for 1 h. Finally, color reaction was performed using TMB substrate (Biolegend, San Diego, CA, USA) and the optical densities were determined by an automated plate reader (HT3, Anthos Mikrosysteme GmbH, Krefeld, Germany). Antibody titers against collagen type II were analyzed by coating Nunc MediSorp ELISA plates (Thermo Fisher Scientific, Waltham, MA, USA) with bovine (MD Bioscience, St. Paul, MN, USA) and murine (ChondrexInc., Redmond, WA, USA) collagen type II at 20 μg/ml each in carbonate/bicarbonate buffer overnight at 4 °C. Sera were applied at a dilution of 1:6000 for 1.5 h at RT and bound antibodies were detected as described for ACPA detection. Antibody titers against *P. gingivalis*, *F. nucleatum*, and *A. actinomycetemcomitans*, bacteria were measured as described by Gemmel *et al*.^36^. In short, 2 × 10^9^ cfu were resuspended in 600 µL carbonate/bicarbonate buffer containing protease inhibitor cocktail (Roche/Sigma Nr: 04693159001) and 1 mM EDTA and were mechanically homogenized 3 times at 6000 rpm for 30 s using the Precellys 24 (Stretton Scientific, Stretton, UK). In between homogenization steps, samples were kept on ice. For the assessment of antibody titers, 100 µL of bacterial lysates were coated on Nunc MediSorp ELISA plates (Thermo Fisher Scientific, Waltham, MA, USA) at protein concentrations of 1 μg/ml in carbonate/bicarbonate buffer overnight at 4 °C. Sera were applied at a dilution of 1:200 for 1.5 h at RT and bound antibodies were detected as described for ACPA detection. A measurement of inflammatory cytokines was performed using the mouse inflammation panel of LEGENDplex (BioLegend, San Diego, CA, USA). For analysis a FACSVerse (Becton, Dickinson and Company, Franklin Lakes, NJ, USA) was used.

### Statistical analyses

The authors declare no restriction of how readers can obtain materials and information. Data sets were analyzed for Gaussian distribution performing Shapiro-Wilk tests. Data following Gaussian distribution were displayed either as means or as dot plots. T-Test (Fig. 2) or One-way ANOVA (Figs 3, 4 and Supplementary Appendix Fig. 2) with post-tests were performed to compare groups. Kaplan-Meier-Estimator was used to display incidence of experimental arthritis using Log Rank (Mantel-Cox), Breslow (Generalized Wilcoxon) and Tarone-Ware-Tests (Fig. 4A). Analysis was performed using Sigma Plot (Version 13.0, Systat Software, Erkrath, Germany) or SPSS (Version 22, IBM, Armonk, NY, USA). Asterisks in Figures denote statistically significant results *p < 0.05; **p < 0.01; ***p < 0.001.

## Electronic supplementary material

supplementary information
