# Supplementary material for: Interplay between P. gingivalis, F. nucleatum and A. actinomycetemcomitans in murine alveolar bone loss, arthritis onset and progression
Source: Sci Rep. 2018 Oct 11;8:15129. doi: 10.1038/s41598-018-33129-z (PMC6181973; doi:10.1038/s41598-018-33129-z)
Supplement: Supplementary file 1 — supplementary information [file 41598_2018_33129_MOESM1_ESM.pdf]

Interplay between *P. gingivalis, F. nucleatum* and *A. actinomycetemcomitans* in murine alveolar bone loss, arthritis onset and progression

- SUPPLEMENTARY INFORMATION -

Meinolf N.B. Ebbers[1], Paul M. Lübcke[1], Johann Volzke[1], Katja Kriebel[2,4], Cathleen Hieke[3], Robby Engelmann[1], Hermann Lang[2], Bernd Kreikemeyer[3] & Brigitte Müller-Hilke[1*]

Supplementary Figure 1

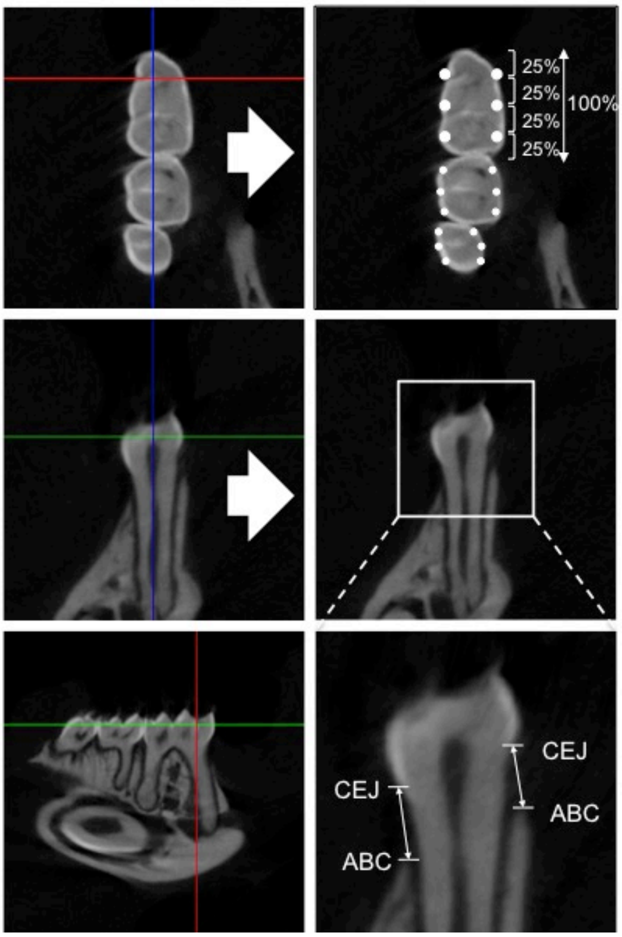

Supplementary Figure 1. Three-dimensional microcomputed tomography (μCT) was used to quantify PD associated bone loss. Alignments illustrate the axial plane of the right hemi-mandible and its three molars (upper left panel). The upper right panel indicates 3 geometrically distributed sites at the lingual and at the buccal sides of each tooth, totaling 18 sites where bone loss was assessed. Middle and lower left panels show coronal and parasagittal planes, respectively. Middle and lower right panels display the alignment used for measuring the distance from the cemento-enamel junction (CEJ) to the alveolar bone crest (ABC).

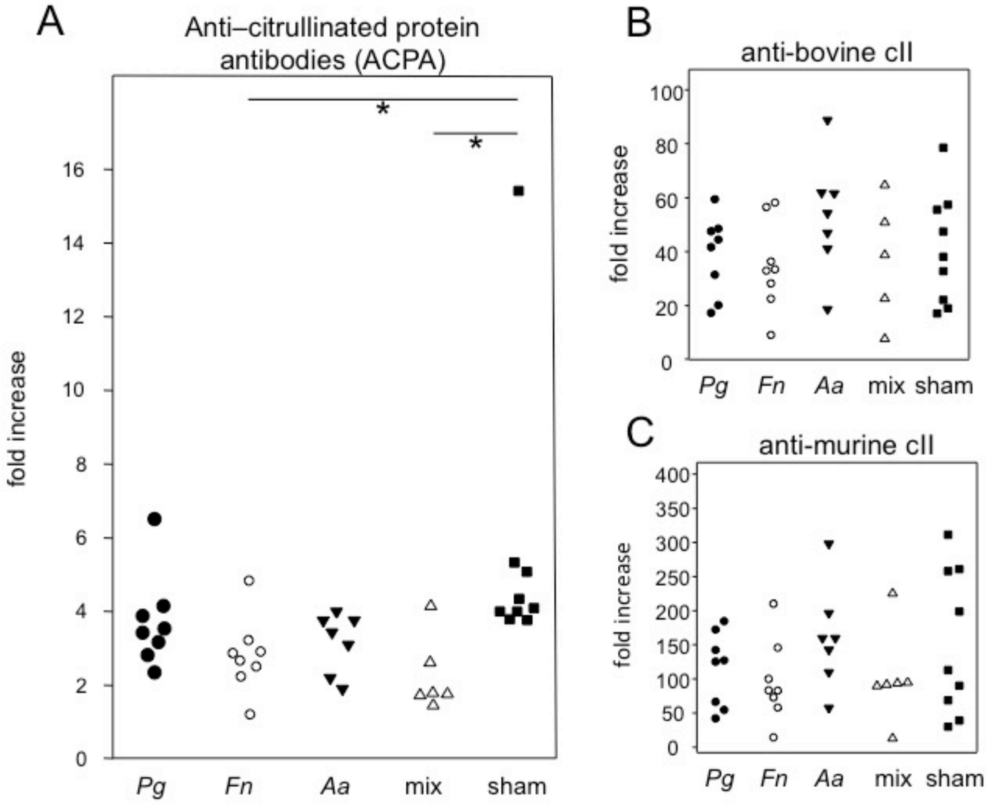

Supplementary Figure 2

Supplementary Figure 2. Anti-citrullinated protein antibodies (ACPA) and antibody titres against bovine and murine type II collagen showed no statistically significant differences at the end of the experimental period. (A) At the end of the experimental period all groups showed equal levels of anti-citrulinated protein antibodies. ACPA were measured by using conventional ELISA. Results are displayed as fold increase comparing individual mice with their control mates. (B and C) All groups also showed equal levels of anti-bovine and anti-murine type II collagen. Abbreviations: *Pg Porphyromonas gingivalis, Fn Fusobacterium nucleatum, Aa Aggregatibacter actinomycetemcomitans,* mix mice inoculated with *Pg* + *Fn* + *Aa,* sham mice inoculated with vehicle, control no treatment at all.

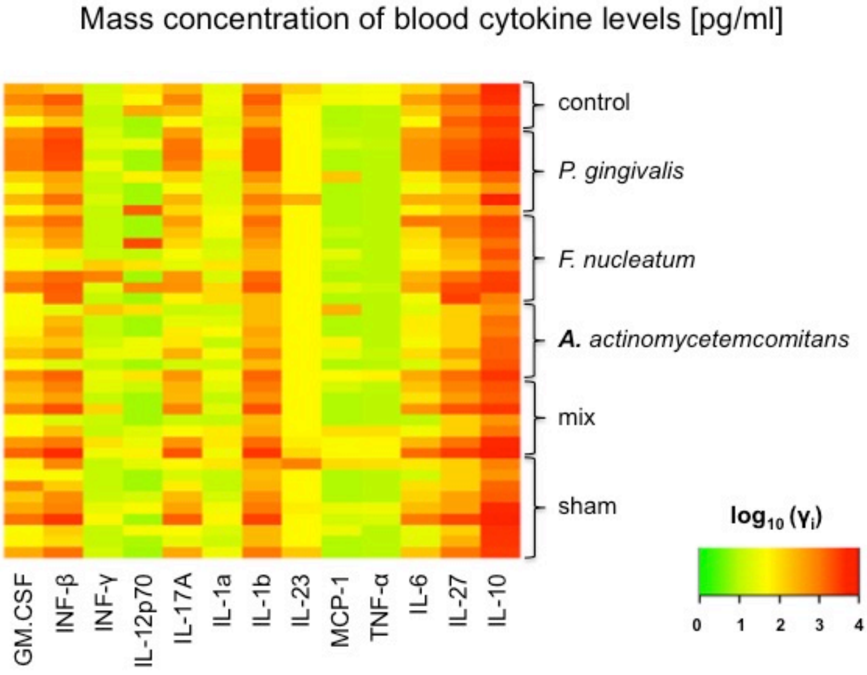

Supplementary Figure 3

Supplementary Figure 3. Mass concentration of blood cytokine levels [pg/ml]. At the end of the experimental period all groups showed equal levels of inflammatory cytokine levels. Statistical analysis showed no significant differences by using hierarchical clustering. Heatmap color key displays the negative decadic logarithm of mass concentration of inflammatory cytokines, which were measured by flow cytometry.
